# Fefferole Flour

**Published:** 1879-08

**Authors:** Marian MacDonald

**Affiliations:** 710 Broadway, New York


### *FEFFEROLE FLOUR.*

This is a cereal flour which the Health Food Company have been experimenting with for a couple of years, and from the use of which, in a multitude of diseased conditions, several patients have derived great benefit. Its great value being demonstrated, it is now offered to the larger public in the belief that it will prove beneficial to a very large class of sufferers at home and abroad.

FEFFEROLE FLOUR is a product of seeds and cereals, and is esssentially nitrogenous in its chemical constitution. It is active and potent as a blood-forming nutriment. It contains a trace of starch, but this starch differs from the starch of grain and potatoes in being non-convertable into glucose or grape sugar by chemical or digestive processes. It is thus a safe and useful food for the diabetic, to whom ordinary starches are little less than active poisons. It is a valuable food for fat people, since its use is quite sure to lesson excess of adipose tissue. It proves an excellent medicinal food in various forms of skin disease, and in the elimination of every kind of blood-poison. In constipation its use is attended with the most gratifying results. Its action is not that of a laxative or cathartic, but rather that of a direct **strengthener of nerve and muscle.** A fleshy lady sends us the following note concerning it:

"I began to use the crackers made by the Health Food Company, of 74 Fourth avenue, New York, from their Fefferole Flour because I was getting too fat, felt sleepy all the time, and couldn't go up stairs without great difficulty in breathing. I have made these crackers my exclusive bread-food for three months, with lean meat, fish, cabbage, greens, etc. I weigh 14 pounds less, can run up stairs without panting, and feel better than I have for years. I was weak, but now I am strong. If any fat person wishes to be safely reduced to symmetry, this food will do it. I tried some so-called 'anti-fats,' but they did me no good. MARIAN MACDONALD.
"710 Broadway, New York."

